# Pre-Crop Chemical Control Has No Effects on Corn Leaf Aphid, *Rhopalosiphum maidis* (Fitch) (Hemiptera: *Aphididae*) Endosymbiotic Bacterial Diversity Along an Industrial Maize Management

**DOI:** 10.3390/insects16040417

**Published:** 2025-04-15

**Authors:** Artúr Botond Csorba, Kálmán Szanyi, Szabolcs Szanyi, Gábor Tarcali, Adalbert Balog, Antal Nagy

**Affiliations:** 1 Department of Horticulture, Faculty of Technical and Human Sciences, Sapientia Hungarian University of Transylvania, Aleea Sighișoarei 2, Târgu Mureș, 540485 Corunca, Romania; 2 Faculty of the Agricultural and Food Sciences and Environmental Management, Institute of Plant Protection, University of Debrecen, Böszörményi Str. 138, 4032 Debrecen, Hungarytarcali.gabor@gmail.com (G.T.)

**Keywords:** intensive management, pesticide application, obligate endosymbionts, facultative endosymbionts, sites

## Abstract

During this research, the corn leaf aphids endosymbiotic bacterial diversity was tested in the same crop systems (monoculture industrial maize as grain for livestock) and the same soil type (Chernozem) when pre-crop pesticide management was used. Bacterial symbionts were analyzed using Illumina systems, and the Silva 16S NR99 V138.2 database was used to assign bacterial taxa on genus and species levels. The presence of the obligate endosymbiont *B. aphidicola* has been clearly detected in all cases, and in all samples but its abundance varied between samples inside crops, but not between crops and generations. The facultative symbionts *S. symbiotica* and *Wolbachia* spp. frequency varied between generations, and increased at generation II; however, differences were not significant. We concluded that the pre-crop pesticide application has no effect on corn leaf aphids bacterial symbionts, so the indirect pesticide application on aphids adaptation is low or nonexistent.

## Simple Summary

In the present study, the endosymbiotic bacterial species diversity was assessed in a conventionally managed maize (*Zea mays*) field, that represents more than 90% of the Central European maize crops. Because no insecticide treatment is allowed legally in Europe to control corn leaf aphids (*Rhopalosiphum maidis*) in industrial maize crops, the only method is the assessment of the pre-crop treatment effects on corn leaf aphids endosymbionts that confers adaptation to host. This is also important because in the last five years, the corn leaf aphid population is increasing; therefore, alternative methods must be considered as no treatments before harvest are allowed in accordance with the European Green Deal. In our study, the primary symbionts *Buchnera aphidicola* as well as a few facultative species (*Serratia symbiotica*, *Wolbachia* spp.) dominate corn leaf aphid communities in all sites and between generations. No effect of pre-crop pesticide treatment on endosymbionts was detected.

## 1. Introduction

Aphids (Hemiptera: *Aphididae*) are one of the most important agricultural pests that cause significant damage due to their feeding on phloem sap, which leads to plant discoloration and deformation. In addition to the direct damage they cause, aphids also serve as vectors for numerous plant viruses of considerable economic importance [1,2].

The management of aphid populations has traditionally been reliant on the application of various classes of insecticides, including pyrethroids, neonicotinoids, carbamates, and organophosphates [3,4]. However, the persistent and widespread use of these chemical control measures over several decades has led to the emergence of resistance in aphid populations, rendering them less susceptible to many of the commonly used insecticidal classes [5,6,7].

The impact of the organophosphorus insecticide, fenitrothion (abbreviated as MEP), on the microbial communities within soils, both with and without a history of insecticide application was examined. The findings indicate that the application of MEP did not result in a significant alteration to the total bacterial population in the soil. However, it was observed to cause a notable reduction in microbial diversity [8]. Furthermore, the application of MEP led to a substantial increase in the relative abundance of MEP-degrading bacteria, particularly those belonging to the genus *Burkholderia*, an endosymbiotic microorganism capable of being acquired from the soil [8,9].

In another study, the bacterial communities present within *Aphis gossypii*, functioning as intracellular symbionts, were analyzed in both naturally occurring and pesticide-treated populations. The results revealed that the composition of the bacterial communities remained stable across all four pesticide-treated samples and most pesticides indicated no influence on the composition of bacterial communities [10].

In the case of fungicide application, the existing hypotheses suggest that certain fungicides possessing antibacterial properties may suppress bacterial endosymbionts within aphids, which play a crucial role in their survival. A study conducted by Evatt Chirgwin et al. [11] investigated this phenomenon and found that the fungicide chlorothalonil did not exert an immediate effect on aphid survival. However, both strobilurin-based fungicides, pyraclostrobin and trifloxystrobin, led to a reduction in aphid survival following 48 h of exposure. Additionally, trifloxystrobin was observed to negatively impact the lifespan and reproductive output of the F1 generation, whereas pyraclostrobin did not exhibit similar effects on these parameters. Despite these findings, none of the fungicides tested were found to consistently influence the population density of the bacterial endosymbionts *Buchnera* or *Rickettsiella* within whole aphid specimens [11].

Previous studies investigating the application of herbicides have emphasized that oxyfluorfen exhibits high persistence and limited mobility in soil. The estimated half-life of oxyfluorfen in soil ranges from 30 to 103 days, depending on the applied dosage and varying levels of precipitation [12]. Given its moderate to high persistence in soil, the potential for environmental contamination with oxyfluorfen is notably increased [13]. Furthermore, recent findings demonstrated that glyphosate treatment led to a significant reduction in the abundance and gene copy number of *Staphylococcus*, while concurrently increasing the abundance and gene copy number of *Enterobacter* in *Harmonia axyridis* [14].

Studies have provided evidence suggesting that symbiotic bacteria (both primary and secondary) may play a significant role in pesticide resistance, primarily due to their capacity to degrade or detoxify various pesticides [15,16]. *Riportus pedestris* has developed resistance to fenitrothion through its symbiont *Burkholderia* [16], while *Plutella xylostella* has been shown to degrade indoxacarb with the assistance of *Bacillus cereus* [17]. In the case of *Bactrocera dorsalis*, the gut symbiont *Citrobacter* plays a key role in the degradation of trichlorfon [15]. Moreover, an increased abundance of *Rickettsia symbionts* in *Bemisia tabaci*, coupled with a reduced presence of *Porteira* and *Hamiltonella*, is correlated with enhanced resistance to thiomethoxam [18].

In aphid populations, exposure to pesticides has been shown to influence the composition of the associated bacterial communities. However, the precise role that these bacterial communities may have in facilitating pesticide resistance remains an area that requires further investigation and a deeper understanding [10].

In the case of *Aphis gossypii*, it has been demonstrated that the yellow morphs of the melon aphid, exhibit significantly higher levels of resistance to commonly used pesticides such as imidacloprid and sulfoxaflor, when compared to the green morphs. The yellow morphs had a higher density of primary symbiont *Buchnera* compared to the green morphs, which can describe a possible relationship between the density of symbiont and pesticide resistance. No significant difference in resistance was observed between the two morphs when exposed to the pesticide avermectin [19].

Facultative (or secondary) symbionts, such as *S. symbiotica*, *H. defensa*, *R. insecticola*, *Spiroplasma*, and *Rickettsia*, can have a detrimental impact on aphid survival and reproduction. However, the extent of this effect appears to be contingent upon both the specific strain of the host and symbiont, as well as the presence or absence of stress factors [20,21]. It has been revealed that aphid lines positive for *Serratia* species were notably more susceptible to insecticide exposure compared to their non-infected counterparts which can be associated with the above-mentioned trade-off [22].

A study investigating the susceptibility of aphids to various insecticides revealed that *Sitobion miscanthi*, harboring *Hamiltonella defensa* exhibited an increased vulnerability to lower concentrations of insecticide, in contrast to their uninfected counterparts [23]. *Sphingomonas*, known for its detoxification capabilities, may present a potential mechanism by which insect pests can metabolize imidacloprid, was demonstrated in this study [24]. *Wolbachia* and *Rickettsia*, both prevalent facultative symbionts in insects, are not only implicated in phenomena such as male killing and parthenogenesis, but they have also been linked to the development of insecticide resistance [25,26]. In the case of the pea aphid, *Acyrthosiphon pisum*, the presence of *Rickettsia* was found to exert influence on several key components of host fitness, including aspects such as body weight and fecundity [27]. In *Serratia* species, it has been observed that these bacteria may harbor plasmids encoding specific enzymes, such as hydrolases, which are believed to be directly involved in the degradation of insecticides [28,29].

The goal of this study was to test the corn leaf aphids endosymbiotic bacterial diversity in the same crop systems (monoculture industrial maize as grain for livestock) and same soil type (Chernozem) when only the pre-crop management differed and used as control. These types of fields represent more than 90% of the Central European maize crops. No previous analyses were made in order to test aphid symbionts under a conventionally management regime, where the pre-crop fields were treated with pesticides. Because no insecticide treatment is allowed legally in Europe to control corn leaf aphids in industrial maize crops, the only methods are to assess the pre-crop (pea in our case) treatment effects on corn leaf aphids endosymbionts.

## 2. Materials and Methods

### 2.1. Study Area and Sampling Methods of Corn Leaf Aphids

**Figure 1:**
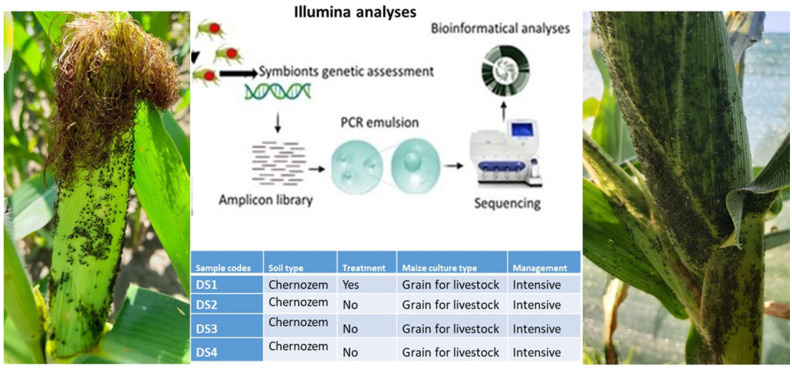
Field collection and genetic analyses of corn leaf aphids under conventionally managed maize fields.

Maize is the largest cash crop in Europe and Hungary. The average corn yield in 2020 was approximately 8.5 million tons and the area dedicated to corn cultivation fluctuated between 778,000 and 906,000 hectares from 2022 to 2024 [30]. In our study, a comprehensive open field aphid assessment and sampling were performed during a 2024 vegetation period, in Eastern and Central Hungary, using four maize fields, all managed under an intensive regime where grain for livestock was produced. The soil type Chernozem was also similar in all fields (Figure 1). From four fields, three (D-A-S2, D-A-S3, D-A-S4) were monoculture for 3–4 years, and one field (D-A-S1) was used as control, where pea was the previous year’s culture and herbicide and insecticide treatments were used during the whole year of production (Table 1).

Inside each of the four fields, two smaller similar plots were defined and sampled. Aphids were collected from maize plants inside these smaller plots to minimize the possible effects of field margins, and the same method of sampling was used in all sites. Apterous viviparous females were searched for inside the colonies, choosing ten maize plants per smaller plot. Inside each colony, the adult and the first instar aphid nymphs (five individuals/plant) were collected and stored in 0.5 mL Eppendorf tubes containing 99% ethanol prior to DNA analysis. The first sampling data were collected on 25 June 2024 and the second on 10 August 2024. In this way, two generations were sampled and analyzed.

### 2.2. Bacterial Symbionts Identification and Bioinformatics Assessments

Aphids bacterial symbionts were analyzed using Illumina systems. From each sample, both adults and the first instar larvae were separately analyzed. In this way, we were able to obtain symbionts from two generations (D1, D2). Illumina PE reads trimmed with cutadapt V3.5 [31]. Untrimmed reads were discarded from the analysis. Sequence level error estimation, filtering, merging, and taxonomy assignment were completed with DADA2 workflow V1.22 [32]. Silva 16S NR99 V138.2 database was used to assign taxa on the species level [33].

### 2.3. Data Analyses

The community composition of the most frequent bacterial symbionts for each aphid colony and site was analyzed by sites and generations (D1, D2) separately. Charts generated with phyloseq V1.38 [34] and polished with ggplot2 V3.5.1 [35] were made and analyzed, and graphical presentation were completed in an R V4.1.2 environment [36]. Alignment of all bacterial genetic sequences was performed using ClustalW, and the results were visualized in EMPeror [37]. Bacterial symbionts frequency (the most dominant 10 taxa) were presented as bar charts. Non-metric multidimensional scaling (NMDS) based on Chao1, Shannon and Simpson diversity were then performed and presented as boxplots. This was performed to compare symbionts diversity by sites and by generations. PERMANOVA analysis of the sample diversities between the groups (sites and generations) was performed in an R V4.1.2 environment [36].

Principal component analyses (PCoA) were used to test the effect of sites and generations on bacterial symbionts abundances for each maize field, where locations (sites) and generations were considered as main components and endosymbiont species diversity as variables. Data analyses and visualization were made using the computer program PAST 4.02. (https://past.en.lo4d.com/windows, accessed on 25 January 2025).

## 3. Results

A high infestation rate of all maize fields with corn leaf aphids was detected during the period of June and August. In certain cases the infestation was 100%, which means that each maize plant had at least one relatively high aphid colony (Figure 1).

**Figure 2:**
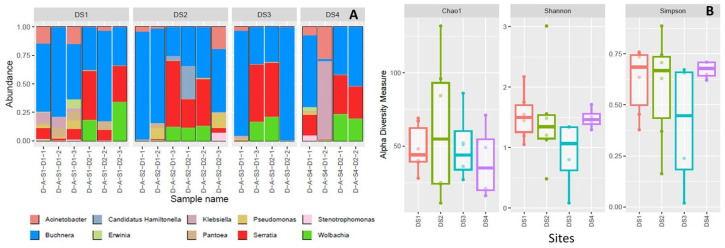
The community composition of the most frequent bacterial symbionts for each aphid colony inside the sites (**A**) and alpha diversity analyses by sites (**B**).

**Figure 3:**
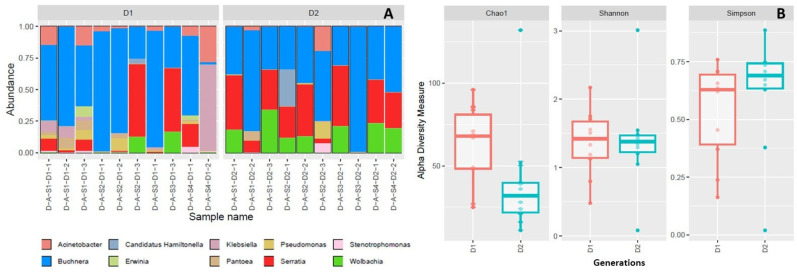
The community composition of the most frequent bacterial symbionts for each aphid colony between generations (**A**) and alpha diversity analyses by generations (**B**).

All sites and samples were dominated by the obligate endosymbiont *B. aphidicola*, this was followed by *S. symbiotica*; however, both *Buchnera* and *Serratia* frequency and diversity varied between sites and generations (Figure 2A and Figure 3A). The third most frequent symbiont was the *Wolbachia* spp., its frequency and diversity also varied between sites and generations. The facultative symbiont *H. defensa* was only present in site 2, while other bacterial taxa frequency (non-symbionts) such as *Acinetobacter*, *Klebsiella*, and *Pantoea* were also higher in some plots and samples (Figure 2A and Figure 3A). Alpha diversity analyses of endosymbionts did not show significant differences between sites nor between generations (Figure 2B and Figure 3B).

**Figure 4:**
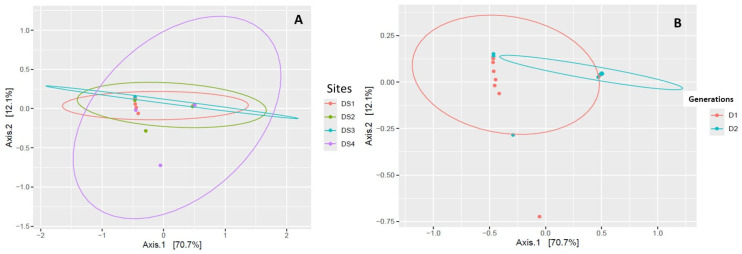
Principal component analyses (PCoA) by sites (**A**) and generations (**B**), where locations (sites) and generations were considered as the main components and endosymbiont species diversity as variables.

Principal component analyses revealed that sites and generations had low effects on corn leaf aphid bacterial symbionts distributions, and the endosymbiont species diversity as variables determined more than 70% of their distributions (Figure 4A,B).

Permanova analysis of the sample diversities between sites (DS1–DS4) and generations (D1 and D2) did not revealed significant differences (Table 2 and Table 3).

## 4. Discussion

In the present study, the population density and diversity of the endosymbiotic bacterial species were assessed in a conventionally managed maize field. These types of fields represent more than 90% of the Central European maize crops. No previous analyses were made in order to test aphid symbionts under a conventionally management regime, where the pre-crop fields were treated with pesticides. Because no insecticide treatment is allowed legally in Europe to control corn leaf aphids in industrial maize crops, the only methods were to assess the pre-crop (pea in our case) treatment effects on corn leaf aphids endosymbionts.

As previous studies mentioned that bacterial endosymbionts are responsible for adaptation, it remains unclear if pesticide applications (even if these are indirect treatments) might or might not have effects on aphids’ population diversity. This question is also important because in the last five years, the corn leaf aphid population has been increasing all over in Europe, and no insecticide application has been made or is under consideration in the EU. It can also be mentioned that in the case of further expansion of this species, alternative methods must be considered as no treatments before harvest will be allowed in accordance with the European Green Deal. In our concrete cases, the primary symbionts *Buchnera* as well as a few facultative species (*Serratia*, *Wolbachia*) dominate corn leaf aphid communities in all sites and between generations.

The presence of the obligate endosymbiont *B. aphidicola* has been clearly detected in all cases, but its abundance varied between samples inside crops, but not between crops and generations. This abundance can be explained because this obligate endosymbiont is required for the survival of aphids and provides essential amino acids that are rare in their phloem sap diet [38].

Other similar studies have also revealed that other novel symbiont can be inherited in aphids body, that physically replace *B. aphidicola* (here in *Geopemphigus* genus)*,* the host may acquire additional symbiont species to adequately provided the essential functions, or even replace the old symbionts with new ones [39]. This change in endosymbionts was previously observed within the tribe *Cerataphidini* (subfamily *Hormaphidinae*) [40,41].

The other dominant symbiont was the *S. symbiotica*, which is well known to be involved in defense against heat and potentially in aphid nutrition. In our study, *S. symbiotica* was present in all sites, both in treated and untreated; however, its frequency highly varied between generations, in generation 1 (D1) from 10 samples only five times were present, and two times in higher abundances, while in generation 2 (D2) from 10 samples nine times were detected, from which seven times in higher abundances. Previous studies also reported that *S. symbiotica* protected pea aphids, *A. pisum*, from heat stress (in both directions) by regulating the aphid metabolome [42]. In our cases the high frequency in generation 2, during the summer period can be an explanation, that *S. symbiotica* indeed conferred adaptation against heat stress, this was clearly detected in aphids new generation abundances during July and August.

The facultative endosymbiont *Wolbachia* spp. was also present in all sites but also in higher frequency in generation 2 (D2). The presence of *Wolbachia* spp. was first reported by Gómez-Valero et al. (2004) in cedar bark aphid (*Cinara cedri cedri* Mimeur, 1936) [43], and it was also detected that *Wolbachia* increased the prevalence of asexual lineages in the aphids. It was also demonstrated that *Wolbachia* spp. can protect aphids (and other arthropods) against fungal pathogens and abiotic stress factors (extreme heat and cold) [44]. Our previous research testing corn leaf aphids symbionts diversity under different climate conditions also revealed that *Wolbachia* spp. was not only present in large-scale field corn crops under warm climates but also under colder conditions, which clearly demonstrates that *Wolbachia* has an important role in the thermal adaptation of corn leaf aphid (authors paper under review).

The endosymbiont *Hemiltonella defensa*, was also detected, but only in one field and in two samples, its role is to protect aphid hosts from parasitoid wasps (in our cases probably against *Aphidius ervi*) and minimizes aphid mortality due to parasitoid attack [45]. It was also demonstrated that the parasitoid wasp *A. ervi* is less attracted to plants infested with aphids harboring *H. defensa* [46].

## 5. Conclusions

In conclusion, we demonstrate for the first time that along a large geographic area and cropping system, the pre-crop pesticide application has no effect on corn leaf aphids bacterial symbionts, so the indirect pesticide application on aphids adaptation is low or inexistent. Earlier studies have demonstrated that several other as fertilizer input, winter temperature, tillage systems but also biodiversity changes as well as different soil management and environmental factors could also influence the expansion of aphids.

As no directed insecticide control as yet exists and is allowed in European maize fields against corn leaf aphids, whereas more than ten synthetic pesticides are available in the USA to combat this pest, before using these compounds (which are all neurotoxins), the effect of symbionts on the distribution and abundance of this aphid species should be considered.

## Figures and Tables

**Table 1:** Different treatments used in the experiment.

Commercial Name	Active Ingredient	Dosage	Time
Basagran 480 SL	bentazone 480 g/L	2 L/ha	3 May 2023
Tropotox XT	MCPB 400 g/L	2 L/ha	3 May 2023
Judo	lambda-cyhalothrin 5 g/L and pirimicarb 100 g/L	1.2 L/ha	15 May 2023
Amistar SC	azoxystrobin 250 g/L	1 L/ha	20 May 2023
Adengo 465 SC	izoxaflutole 225 g/L, thiencarbazone-methyl 90 g/L and cyprosulfamide 150 g/L	0.4 L/ha	22 April 2024

**Table 2:** PERMANOVA analysis of the sample diversities between Groups DS1 and DS2 (A), Groups DS1 and DS3 (B), Groups DS1 and DS4 (C), Groups DS2 and DS3 (D), Groups DS2 and DS4 (E) and Groups DS3 and DS4 (F), in a site sample feature.

**A**	**Df**	**SumsOfSqs**	**MeanSqs**	**F.Model**	**R2**	**Pr(>F)**
Soil Type	1	0.18	0.18	0.56	0.05	0.41
Residuals	10	3.17	0.31	NA	0.94	NA
Total	11	3.36	NA	NA	1.00	NA
**B**	**Df**	**SumsOfSqs**	**MeanSqs**	**F.Model**	**R2**	**Pr(>F)**
Soil Type	1	0.13	0.13	0.43	0.05	0.60
Residuals	8	2.46	0.30	NA	0.94	NA
Total	9	2.60	NA	NA	1.00	NA
**C**	**Df**	**SumsOfSqs**	**MeanSqs**	**F.Model**	**R2**	**Pr(>F)**
Soil Type	1	0.29	0.29	0.87	0.09	0.38
Residuals	8	2.67	0.33	NA	0.90	NA
Total	9	2.96	NA	NA	1.00	NA
**D**	**Df**	**SumsOfSqs**	**MeanSqs**	**F.Model**	**R2**	**Pr(>F)**
Soil Type	1	0.05	0.05	0.15	0.01	0.89
Residuals	8	2.70	0.33	NA	0.98	NA
Total	9	2.75	NA	NA	1.00	NA
**E**	**Df**	**SumsOfSqs**	**MeanSqs**	**F.Model**	**R2**	**Pr(>F)**
Soil Type	1	0.16	0.16	0.45	0.05	0.63
Residuals	8	2.90	0.36	NA	0.94	NA
Total	9	3.07	NA	NA	1.00	NA
**F**	**Df**	**SumsOfSqs**	**MeanSqs**	**F.Model**	**R2**	**Pr(>F)**
Soil Type	1	0.20	0.20	0.54	0.08	0.52
Residuals	6	2.19	0.36	NA	0.91	NA
Total	7	2.39	NA	NA	1.00	NA

**Table 3:** PERMANOVA analysis of the sample diversities between generations D1 and D2 in a time sample feature.

	Df	SumsOfSqs	MeanSqs	F.Model	R2	Pr(>F)
Time	1	1.09	1.09	4.13	0.18	0.02
Residuals	18	4.78	0.26	NA	0.81	NA
Total	19	5.88	NA	NA	1.00	NA

## Data Availability

The datasets used and/or analyzed during the current study available at figshare: https://figshare.com/articles/dataset/Corn_Leaf_aphid_symbionts_genetic_analyses/25027973, accessed on 31 January 2025.
